# Aspects of tree shrew consolidated sleep structure resemble human sleep

**DOI:** 10.1038/s42003-021-02234-7

**Published:** 2021-06-11

**Authors:** Marta M. Dimanico, Arndt-Lukas Klaassen, Jing Wang, Melanie Kaeser, Michael Harvey, Björn Rasch, Gregor Rainer

**Affiliations:** 1 Department of Neuroscience and Movement Sciences, Section of Medicine, University of Fribourg, Fribourg, Switzerland; 2 Department of Psychology, University of Fribourg, Fribourg, Switzerland; 3 Department of Neurobiology, School of Basic Medical Sciences, Nanjing Medical University, Nanjing, China

**Keywords:** Neuroscience, Psychology

## Abstract

Understanding human sleep requires appropriate animal models. Sleep has been extensively studied in rodents, although rodent sleep differs substantially from human sleep. Here we investigate sleep in tree shrews, small diurnal mammals phylogenetically close to primates, and compare it to sleep in rats and humans using electrophysiological recordings from frontal cortex of each species. Tree shrews exhibited consolidated sleep, with a sleep bout duration parameter, τ, uncharacteristically high for a small mammal, and differing substantially from the sleep of rodents that is often punctuated by wakefulness. Two NREM sleep stages were observed in tree shrews: NREM, characterized by high delta waves and spindles, and an intermediate stage (IS-NREM) occurring on NREM to REM transitions and consisting of intermediate delta waves with concomitant theta-alpha activity. While IS-NREM activity was reliable in tree shrews, we could also detect it in human EEG data, on a subset of transitions. Finally, coupling events between sleep spindles and slow waves clustered near the beginning of the sleep period in tree shrews, paralleling humans, whereas they were more evenly distributed in rats. Our results suggest considerable homology of sleep structure between humans and tree shrews despite the large difference in body mass between these species.

## Abstract (web summary)

Dimanico et al investigated sleep in tree shrews using electrophysiological recordings and compared it to equivalent read-outs in rats and humans. They reported that there was considerable homology of sleep structure between humans and tree shrews despite the difference in body mass between these species.

## Introduction

Sleep is a universal phenomenon among mammalian species. It is notable that different species exhibit large differences in various aspects related to sleep, for example in terms of overall sleep duration or in terms of how sleep is distributed over 24 h^1^.

Sleep encompasses two distinctly separable stages, which are rapid eye movement (REM) sleep and non-REM (NREM) sleep. NREM sleep is further fractionated into three separable substages, at least in humans. The sleep stages are defined according to electrophysiological aspects, such that NREM1 defines the lightest sleep and its onset is marked by increased theta activity. NREM2 follows NREM1 and is characterized by the occurrence of K-complexes (KC, <1 Hz) and sleep spindles (9–16 Hz)^2,3^. Overall, low frequency, large amplitude activity keeps increasing during a sleep cycle, culminating in NREM3, the stage with the largest delta (δ) activity (0.5–4 Hz) and prominent slow waves, which for this reason is also referred to as SWS (slow-wave sleep). During REM sleep, the cortical EEG exhibits high frequency, low amplitude, desynchronized oscillatory activity that bears similarities to the EEG during wakefulness, while however being accompanied by muscle atonia and eye movements^4,5^. Sleep stages alternate cyclically throughout the night, with one cycle usually identified as a NREM episode followed by a REM episode and a single cycle lasting around 90 min in humans.

Elucidating how sleep is regulated and maintained, and relating it to other aspects of brain function such as memory formation requires work in animal models. Much of this work has been done in rats and mice, and has produced important insights into how sleep is regulated and maintained in these rodent species. For example, it has been shown that neural activations encountered during the awake state recur during subsequent sleep periods, and this “replay” is thought to contribute to long-term memory consolidation^6,7^. Other work has revealed that transitions between behavioral states are under the control of brain stem or basal forebrain centers, such that for example activation of circuits in the medulla can serve to initiate REM sleep^8^. However, in this context it is relevant that sleep in rodents displays substantial differences from human sleep. Most strikingly, while human sleep is largely monophasic and consists of essentially a single sleep period, sleep in rodents is highly fractionated such that sleep in these species is often interrupted by periods of wakefulness. In addition, while NREM sleep in humans consists of three substages, there is only one main discernible NREM sleep stage in rodents, although there is evidence also in rodents for further fractionation of NREM sleep that is however more challenging to detect given the overall shorter sleep cycle duration in this species,^9,10^. Finally, the main sleep period of rats and mice is daytime, while humans sleep during the night, indicating that the coupling of sleep to circadian rhythms must involve differential regulation of neural circuits and associated biochemical pathways.

The discrepancy regarding the main sleep period between humans and laboratory rodents has led researchers to study several mammalian species, whose sleep patterns more closely approximate those of humans. One of these diurnal species is the Sudanian grass rat (*Arvicanthis ansorgei*), a rodent species that spends on average about 50% of the day in the awake state compared to 30% awake time during the night^11^. *Arvicanthis* are indeed also crepuscular, and are awake over 80% of the time around transitions between day and night, an attribute that is shared also by other rodent species^12^. Overall, the sleep pattern of *Arvicanthis*, while being more diurnal in nature than laboratory rodents, remains nevertheless substantially fragmented and in this respect still far from human sleep. Another interesting species from a sleep perspective is the four-striped grass mouse (*Rhabdomys pumilio*), a mouse species that also exhibits pronounced crepuscularity^13^. Interestingly, *Rhabdomys* appear to be highly sensitive to the rate of luminance change around day/night transitions, such that a gradual illumination change over a 6 h transition period tends to further enhance diurnal locomotor activity patterns. These two diurnal rodent species are examples of less frequently used animal models, which nevertheless provide important comparative perspectives on mammalian sleep.

In the present study, our goal is to study sleep patterns in tree shrews (*Tupaia belangeri*), a small diurnal mammalian species native to south-eastern Asia, which belongs to the order *Scandentia* making them, next to the flying lemurs, the closest living relatives of primates,^14^. Their day active lifestyle is consistent with a large and highly differentiated visual cortex, which exhibits a number of striking similarities to the visual cortex of primates^15,16^. Tree shrews are adept at learning a variety of tasks. For example, they can acquire a rule-based spatial task, selecting always the left of two available arms in a multiple arm maze, and apply this rule to novel problems not part of the training set^17^. Furthermore, they exhibit rapid learning in a visuospatial search paradigm where animals must search for rewards hidden below visual cues^18^. Consistent with this observation, we have recently shown that tree shrews rapidly learn a visual discrimination task, indeed showing parallels to non-human primates (*macaca mulatta*), particularly during initial task acquisition with high contrast targets^19^. Memory encoding over 24 h periods has been investigated in tree shrews in two studies that employed the novelty preference paradigm^20,21^, where tree shrews exhibited novelty preference in both novel object and location conditions following 24-h retention periods, suggesting robust long-term memory encoding of objects and their locations during habituation sessions. Note that in these studies, tree shrews performed the task during their peak activity period, i.e., the light period corresponding to daytime, where animals would normally interact with their environment. Interestingly, rats often fail to display novelty preference over such long intervals^22–25^; but note that much longer retention times have been observed using alternative training procedures^26,27^.

While relatively little is known so far about tree shrews sleep, the pioneering work of Coolen and colleagues has shown that during the dark, inactive phase, members of this species spend a large fraction of the time asleep (over 90%)^28^. In fact, during the night tree shrews tend not to venture outside their nest box in laboratory housing (our own observations), or their nest in the wild^29^, as their retinae are almost exclusively composed of cones^30^, which do not function under low illumination. This consolidated sleep pattern of the tree shrew appears more akin to the monophasic sleep of humans than the fragmented sleep of rodent species. It is indeed possible that this absence of rod photoreceptors may have played a role in the establishment of monophasic sleep in diurnal tree shrews. The main goal of the present study is thus to provide a comprehensive characterization of tree shrew sleep cycles and their progression throughout the sleep period. Sleep data from different species can be difficult to compare, because experimental work in different laboratories often entails technical and methodological differences that complicate direct comparison. Here, we therefore collected data also from rat and human subjects, which we then analyzed using similar methods to facilitate direct comparison. We expand our analyses to sleep spindles, which occur during NREM sleep coupled to slow waves of the EEG or LFP signals in humans^31–33^ as well as rodents^34–38^. Spindle activity is burst-like, occurs in the 9–16 Hz frequency band and can readily be recorded in frontal cortical areas including the anterior cingulate cortex (ACC).

Here we find that, despite their small size, tree shrew sleep parameters were more similar to humans than were the rats. Namely, tree shrews demonstrated a more consolidated sleep structure, their sleep contained an intermediate NREM stage and like humans, but not rats, displayed sleep spindle and slow wave coupling events that were clustered near the beginning of the sleep period.

## Results

We recorded local field potentials (LFPs) from the ACC of rats (*n* = 5), and tree shrews (*n* = 4) during sleep sessions lasting eight consecutive hours. In rats, recordings were made in a home cage-like environment that allowed for tethered recordings during the natural sleep period. Tethered recordings were necessary for the rat as they participated in a subsequent experiment. As tree shrews sleep in a small wooden nest box, recordings were made by the use of a wireless head stage. In humans, (*n* = 5), we made nightly sleep recordings using standard EEG electrodes in a sleep laboratory setting.

**Fig. 1:**
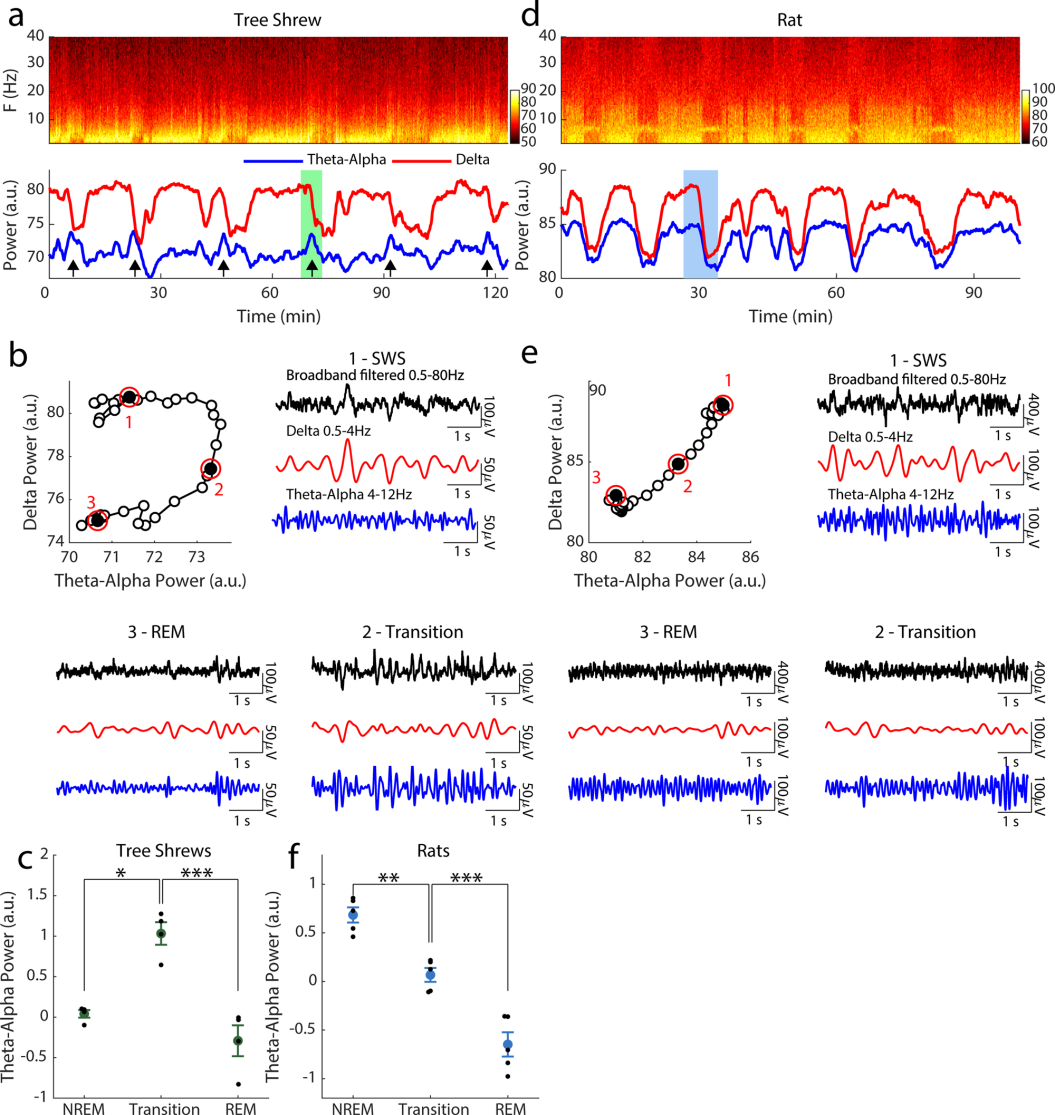
Dynamics of the transition episodes in tree shrews and rats. Spectrograms of example segments of tree shrew (**a**) and rat (**d**) sleep, with the corresponding delta power, red (0.5–4 Hz) and theta-alpha power, blue, (4–12 Hz) shown below. Black arrows indicate periods of increased theta-alpha power accompanying NREM/REM transitions. Selected transitions for tree shrew and rats are shown in (**b**) and (**e**) respectively. Top level panels show delta vs. theta-alpha power for the sleep segments highlighted in (**a**) and (**d**). Circled epochs 1–2 are expanded in the surrounding numbered panels, showing the corresponding filtered LFP traces. Note that in the example rat delta and theta-alpha power decrease together while in the tree shrew an initial decline in delta power is accompanied by a transient increase at the theta-alpha band, see arrows in (**a**). Indeed this transient increase in theta-alpha power during transitions was significant for the population of tree shrews (*n* = 4) as shown in (**c**), while theta-alpha significantly decreased during transitions in rats (*n* = 5) as shown in (**f**), paired *t*-tests, *p < 0.05, **p < 0.01, ***p < 0.001. Error bars reflect the standard error of the mean. Color bars reflect Fourier power in arbitrary units.

An ACC LFP spectrogram segment from an example tree shrew is shown in Fig. 1a. Several sleep cycles composed of high delta activity (0.5–4 Hz), corresponding to NREM episodes, are followed by shorter periods of low delta activity, corresponding to REM epochs. We noticed that ACC LFP power in the theta-alpha range (4–12 Hz) reliably exhibited notable peaks during the NREM to REM transitions, i.e., during the downward slope of delta power. To characterize the NREM to REM transition, we plotted delta power vs. theta-alpha power around the time of a typical transition (see Fig. 1b), illustrating that transient enhancements in theta-alpha power occur specifically during the sleep state transition, i.e., accompanying intermediate values of delta power. The broadband (0.5–80 Hz), as well as delta and alpha-theta band filtered, LFP time courses are shown for example segments of NREM transition and REM states. Intermediate delta power, as well as prominent theta-alpha oscillations are readily apparent for this example transition state, and indeed this pattern was reliably observed in all tree shrews investigated (see Fig. 1c). For this analysis, we identified the center of the transition state as the point of minimum first derivative in delta power, and then estimated average alpha-theta power in a 10 s segment around this time point. We compared this value to the theta-alpha values during preceding NREM sleep and following REM states also estimated for 10 s segments. No similar transition state was observable in the rat, as suggested by the example spectrogram and LFP power time courses shown in Fig. 1d. Here, theta-alpha and delta power exhibited highly correlated time courses, such that NREM to REM state transitions were accompanied by reductions in both of these LFP frequency bands. An analysis of an example transition (see Fig. 1e) illustrates the lack of a transition state; an observation that is validated for all rats as shown in Fig. 1f. Note that in the rat, narrow theta band (6–7 Hz) activity can be seen in the example spectrogram, as well as in the LFP trace during REM. We present evidence for an intermediate sleep stage that prominently occurs in the tree shrew at transitions between NREM and REM sleep. This sleep stage is characterized by intermediate delta power as well as enhanced theta and alpha waves, and thus shares the spectral signatures of the NREM1 sleep stage in humans that accompany transitions from wakefulness to slow-wave sleep. Taken together, our data suggest that tree shrew NREM sleep consists of two separable stages, an NREM sleep stage, and an intermediate (IS-NREM) stage that occurs as the brain state transitions from NREM to REM. For rats, no intermediate stage was discernible in our recordings, but previous work has identified this kind of activity (see “Discussion”).

**Fig. 2:**
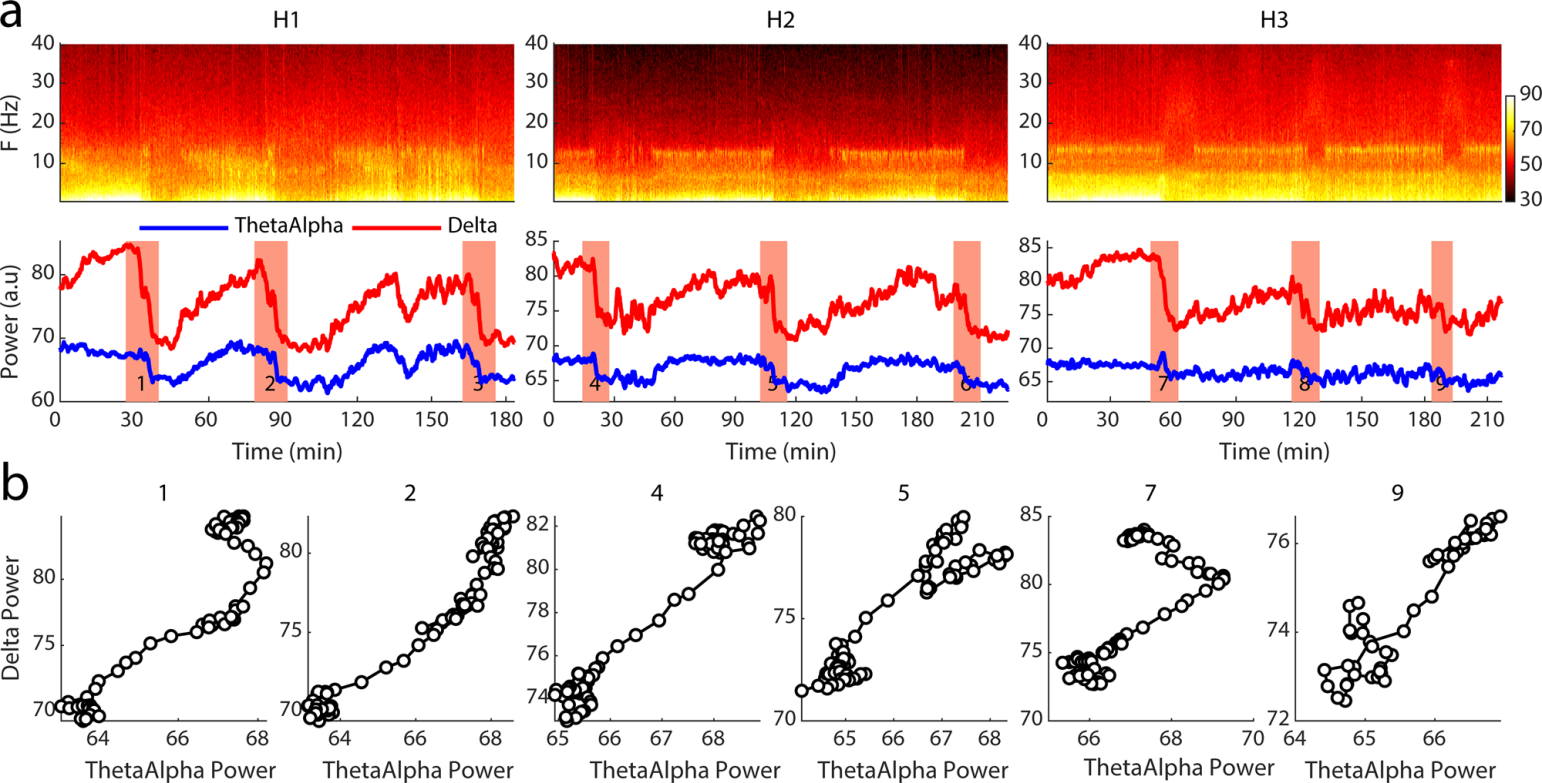
Example transition episodes in humans. **a** Top, spectrograms of example episodes of human sleep from three subjects. Bottom panel, corresponding traces of delta (0.5–4 Hz) and theta-alpha (4–12 Hz) power in red and blue respectively. Red patches highlight transition segments from NREM with high delta power to REM sleep with low delta power. **b** Delta power vs. theta-alpha power is shown for six example transitions. Title numbers corresponding to transitions with the same numbers from (**a**). Note theta-alpha power increases over the time course of a few transitions (see transition 1 and 7) in comparison to other transitions in which a simultaneous decrease of delta and theta-alpha power is observable. Color bars reflect Fourier power in arbitrary units.

The NREM1 sleep stage in humans is a transition sleep stage that is also related to fluctuations in alpha as well as theta power in the EEG signal, but these are typically not as conspicuous as the ones we have seen in tree shrew LFP signals. Nevertheless, we examined the transition from NREM to REM in human subjects using a similar approach previously explained for the small mammals above. In this case, we visually inspected the traces in humans and selected all episodes at the transition from NREM to REM. Example transitions are shown in Fig. 2a, showing spectrograms and filtered EEG time courses from three human subjects. Figure 2b shows example trajectories in delta vs. theta-alpha activity, illustrating that in the majority of cases, activity in these frequency bands was highly correlated, resembling coherent reductions in both frequency bands during transitions as we had observed in rat LFP. However, intriguingly we did observe on multiple occasions a transient enhancement of theta-alpha activity during the transition, which in turn resembles our findings in tree shrew although the theta-alpha peaks were more variable and less pronounced in humans. Considering the total number of transitions observed in our human subjects (*n* = 5), we document 5/21 (24%) of transitions involved theta-alpha peaks, whereas 76% were of correlated nature with coherent reductions in delta and theta-alpha activity. This pattern of results suggests that among the three species investigated, we observed a notable homology in terms of electrophysiological signatures of NREM to REM transitions between tree shrews and humans.

**Fig. 3:**
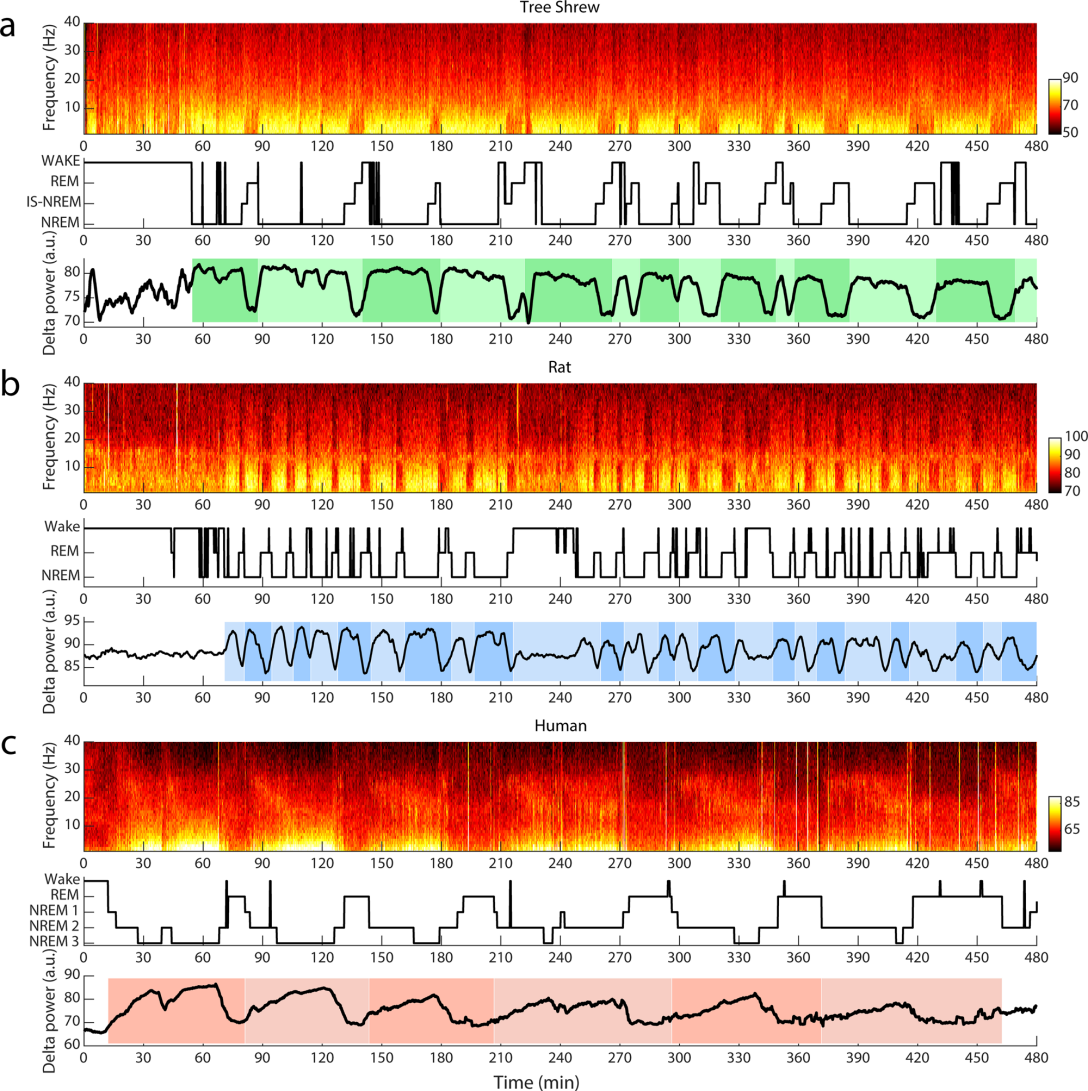
Example sleep recording of tree shrews, rats, and humans. **a** Top, spectrogram of an 8 h recording period for an example tree shrew. Middle, hypnogram showing NREM sleep, IS-NREM as well as REM and Wake. Bottom panel, the corresponding delta power (0.5–4 Hz) trace with colored patches indicating sleep cycles, and changes in shading indicating independent sleep cycles. (**b**) and (**c**) are as (**a**) but for example rat and human respectively. Note, correspondence of sleep cycles and delta power fluctuations across species. Color bars reflect Fourier power in arbitrary units.

We proceeded to examine entire sleep sessions in each of the species, taking into account the two separable tree shrew NREM stages we have identified above. An ACC LFP spectrogram for a tree shrew 8 h sleep session is shown in Fig. 3a (top panel), demonstrating sleep cycles comprising prolonged epochs of NREM with high delta power that could last up to about 30 min. Using manual sleep scoring, and taking into account micro-movements of the animals, we generated a hypnogram as shown in Fig. 3a (middle panel). The sleep cycles, i.e., systematic progression from NREM to IS-NREM to REM sleep stages, as well as sometimes wakefulness, are readily apparent. We note that the sleep cycles were largely well captured by the time course of delta-power (0.5–4 Hz) in the ACC LFP (see Fig. 3a, bottom panel). Rat sleep architecture differed markedly from that of tree shrew, as shown in Fig. 3b. The ACC LFP spectrogram, as well as the hypnogram for this sleep period, exhibited periods of NREM sleep that appeared to be of considerably shorter duration than in tree shrew, often lasting <10 min, and punctuated by frequent episodes of wakefulness. As was the case for tree shrews, ACC delta-power largely captured the more fragmented nature of sleep in the rat, characterized by more frequent cycling between sleep states (see Fig. 3b, bottom panel). For comparison with these data from two small mammal species, we used frontal EEG recordings obtained from human subjects recorded in a sleep laboratory setting. An example spectrogram and hypnogram, shown in Fig. 3c, reveal typical progression between sleep states observed in humans, in particular contiguous periods of elevated delta-power, corresponding to NREM sleep, as well as concomitant REM periods and occasional short epochs of wakefulness. As for the small mammals, delta-power provided a good estimate of the sleep cycle in humans (see bottom panel).

**Fig. 4:**
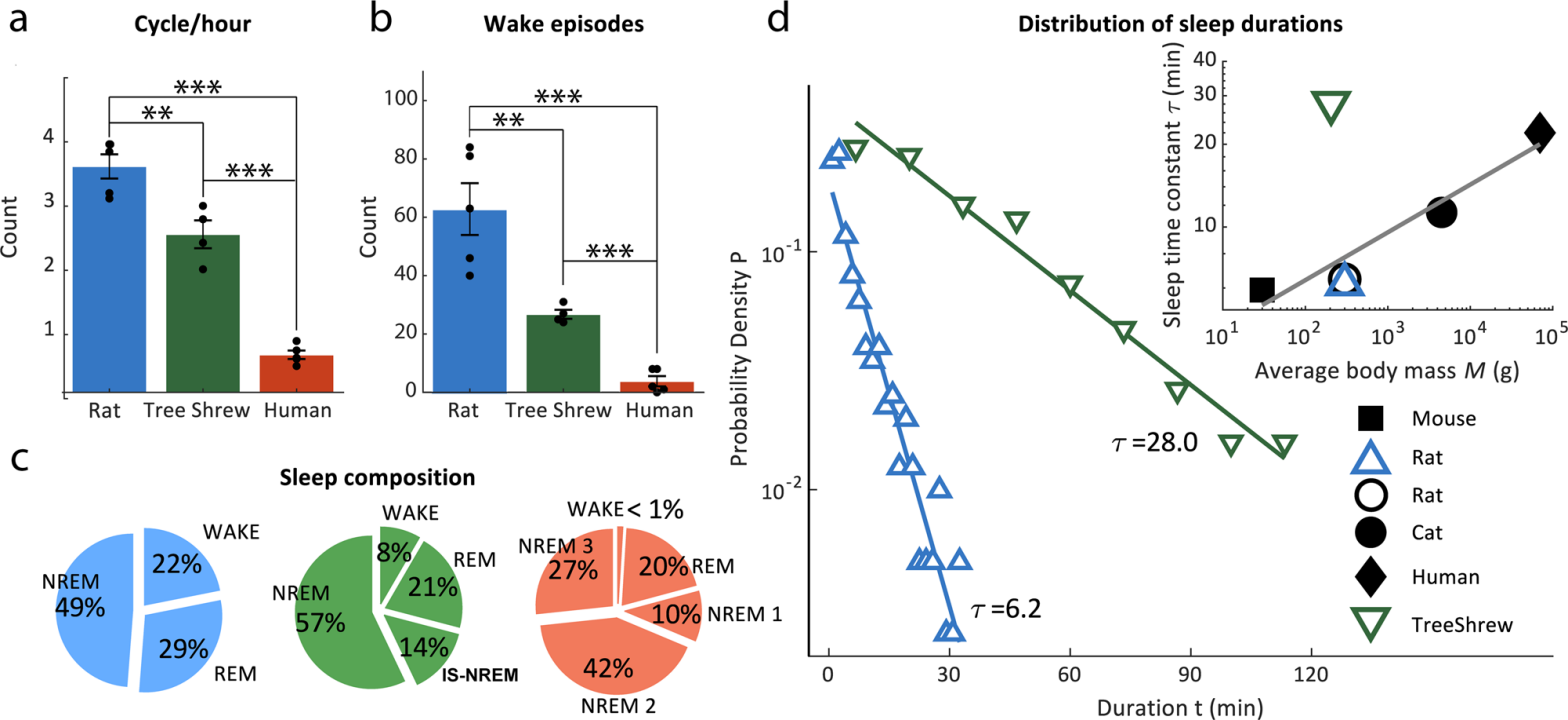
Tree shrews have significantly longer and less fragmented sleep than rats. **a** Bar chart is showing average count of sleep cycles per hour across species, Rats *n* = 5, tree shrews *n* = 4, humans *n* = 5. Note, significantly more sleep cycles per hour in rats in comparison to tree shrews. **b** Bar plot is illustrating the mean count of wake bouts over the course of the recording period across species. Rats have significantly more wake episodes than tree shrews. **c** Pie charts showing the mean fractions of different behavioral stages after sleep onset in tree shrews, rats, and humans respectively. **d** Distributions of sleep bout durations for rats and tree shews. The data of both species follows a power law function with different time constants, *τ*. Lines between data points indicate the fit of the function for tree shrew and rat in green and blue respectively. The inset shows values of the time constant *τ* for the distribution of sleep duration in relation to the body mass of different species. Human, mouse, rat, and cat data, black markers were taken from a previous publication^34^. Note that values obtained here for the rat match those of the previous study. Tree shrew and rat *τ* values are added based on our own data and analyses. Error bars reflect the standard error of the mean.

The example recordings presented above highlight some key differences and similarities of the sleep structure across species, such that spectrograms displayed periods of prominent low-frequency delta power related to the occurrence of NREM sleep. Whereas rat sleep was highly fragmented and frequently punctuated by wake episodes, human sleep exhibited lengthy sleep cycles dominated by NREM sleep. Tree shrew sleep structure was more similar to that of humans in terms of fragmentation. Quantifying these effects across all of the recordings in each species (see Fig. 4a), we found that rats exhibited a significantly higher number of sleep cycles per hour compared to tree shrews (cycles/hour rat: 3.5 ± 0.2 SEM, tree shrews: 2.5 ± 0.2 SEM, unpaired *t*-test, *p* = 0.009, *t* = 3.5) consistent with the idea that tree shrew sleep is significantly less fragmented than that of the rat. Our analyses also show that humans possess the longest sleep cycle of these three species (0.6 ± 0.07 SEM cycles per hour), corresponding to a typical cycle duration of ~1½ hours. We also examined the amount of wakefulness present in each species during the sleep period (see Fig. 4b). Rats exhibited a significantly elevated number of wake episodes throughout the sleep period compared to tree shrew, with both small mammals exhibiting more frequent awakening compared to humans (average number of wake episodes rats per sleep period: 62.8 ± 8.9 SEM, tree shrews: 27.6 ± 1.5 SEM, humans: 3.8 ± 1.7 SEM, (unpaired *t*-test: rats vs tree shrews, *t* = 3.6, *p* = 0.009; rats vs humans, *t* = 14.7, *p* < 0.001; tree shrews vs humans *t* = 9.9, *p* < 0.001). On a related note, we computed the fraction of time spent in NREM, REM, and wakeful state for the whole recording period after sleep onset (Fig. 4c). Notably, rats were awake during about 22% of the recording, a significant increase over both tree shrews and humans (8% and 1% respectively, *χ*2-test: *χ*2 = 10.1, *p* < 0.0001). The amount of REM was similar across species. Taking all of the NREM stages together, rats spent less time in this sleep stage (49%) than tree shrews and humans (71%, 79% respectively *χ*2-test: *p* < 0.01). Furthermore, tree shrews and humans exhibited similar durations of NREM sleep (cumulated NREM2 and NREM3 stages), corresponding to 57% and 69% respectively of the sleep cycle (*χ*2-test: *χ*2 = 3.1, *p* = 0.08), and IS-NREM (14% and 10% respectively, (*χ*2-test: *χ*2 = 0.75, *p* = 0.38).

A pertinent characteristic of mammalian sleep is the systematic relationship between the duration of sleep episodes and the probability of their occurrence^39^, such that longer sleep episodes occur less frequently than shorter ones. A similar relationship holds for the duration of wake periods that occur during the sleep session, and both systematic relationships can be described by the time constants *α* for wake periods and τ for sleep durations. Interestingly, time constant α has been shown to be invariant at *α*≈2.2 across numerous mammalian species for which data are available, including mouse, rat, cat, and human. Here, we determined the value from a linear fit between wake episode duration and occurrence probability on a log-log scale for rat and tree shrew for our dataset. We estimate values of *α* = 2.3 ± 0.2 (SEM) for the rat and *α* = 2.1 ± 0.3 (SEM) for the tree shrew, and these values fall in the range of α parameters previously reported for four mammalian species including humans^39^. For the sleep bout duration parameter τ, we estimated the probability density for different sleep bout durations in the datasets for rat and tree shrew, as shown in Fig. 4d. For the rat, we estimated τ = 6.2, corresponding closely to previous results; whereas for tree shrews we determined τ = 28.0, a value that even exceeds the reported parameter for humans (τ = 22.0). Note that these estimates are based on pooling sleep bout durations from all subjects in each species. Considering each individual subject, we find closely corresponding values of τ = 6.3 ± 1.1 (SEM) and τ = 25.7 ± 1.9 (SEM) for rats and tree shrews respectively. For illustration, Fig. 4d inset, we plot τ values against body mass for tree shrew as well as values for other mammalian species taken from a previous report^39^, emphasizing both a wide divergence of τ, particularly considering mammals of similar body mass, as well as a close correspondence between the values obtained for the rat in our study and previous literature.

**Fig. 5:**
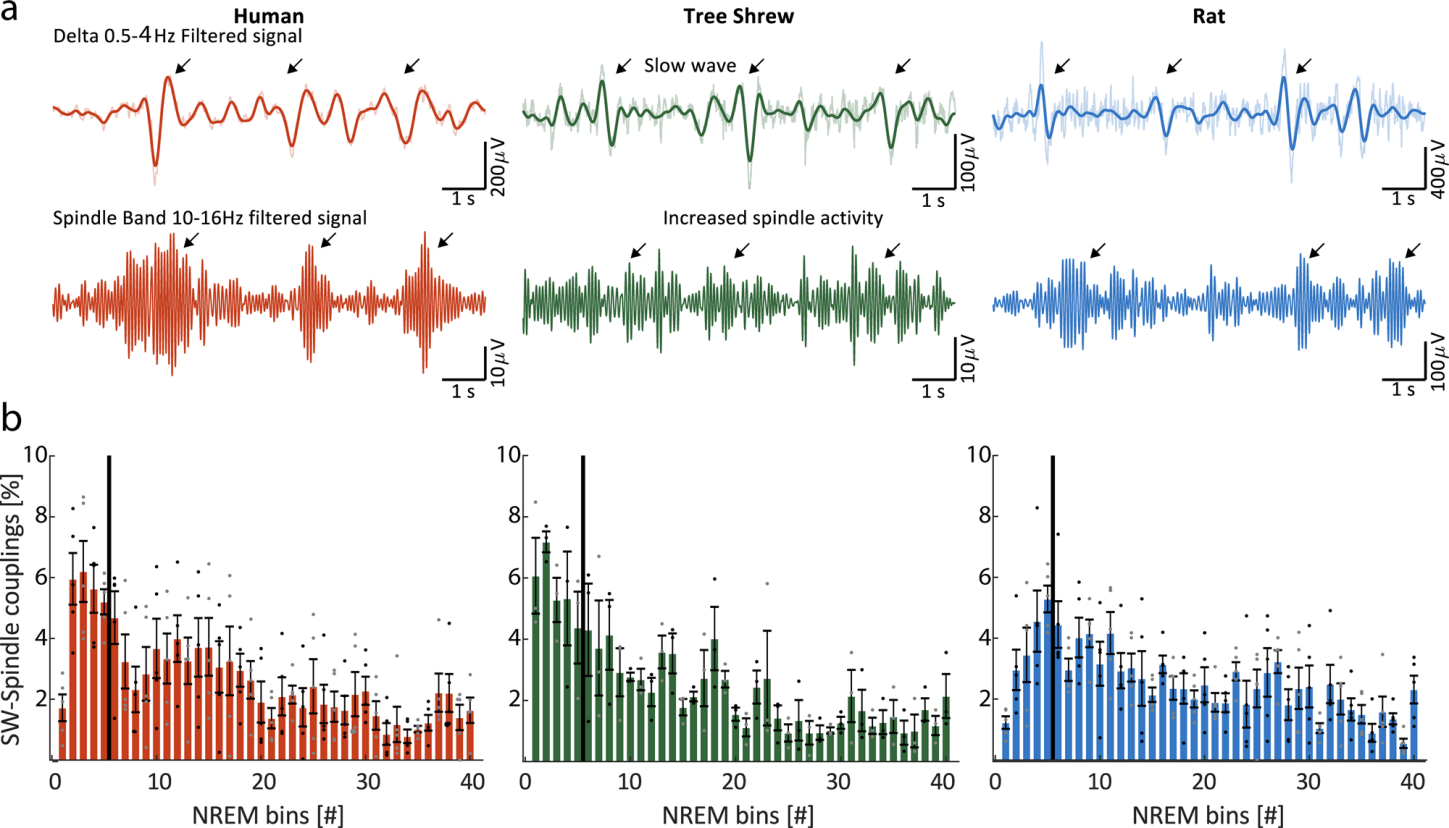
Slow wave and sleep spindle analyses. **a** Example traces of NREM for the different species showing both slow waves and spindle band activity. Top row, signal filtered in the delta range (0.5–4 Hz) superimposed upon the wideband, shaded area, (0.5–45 Hz) signal. Arrows denote slow wave events. Bottom row, the same signal, but filtered in the spindle range (10–16 Hz), with arrows indicating spindle events. Note the co-occurrence of slow waves and bursts of spindle band activity. **b** Group averaged distribution of the coupling event across species, rats *n* = 5, tree shrews *n* = 4, humans *n* = 5. Histograms show the group average distribution of slow wave-spindle coupling events in percentages over the time course of NREM sleep divided in 40 bins per recording (error bars indicate ±SEM). Note the black line represents the section of the first five bins containing coupling events during the beginning of the sleep period that were used for statistical comparison. Error bars reflect the standard error of the mean.

**Fig. 6:**
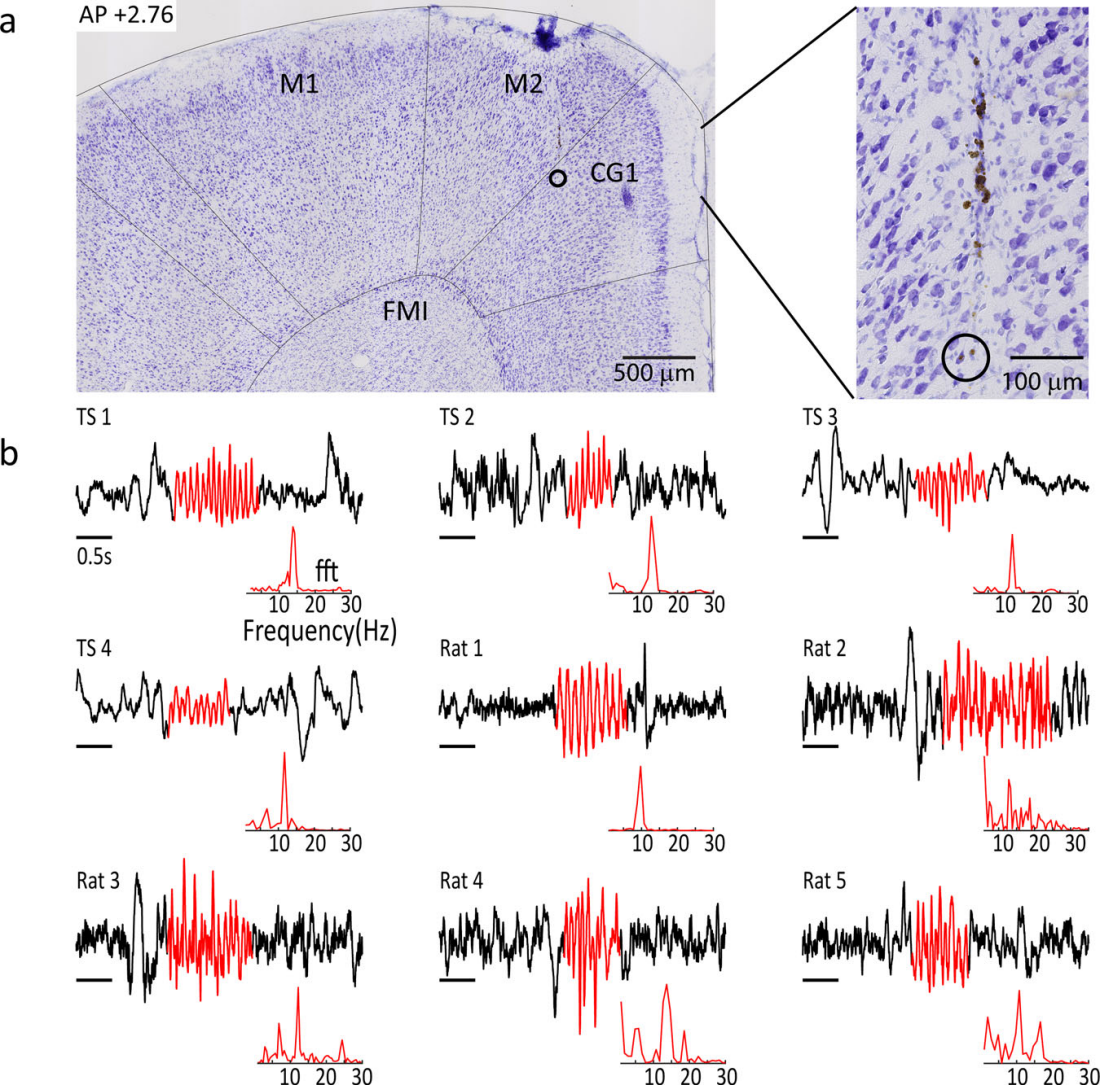
Validation of recording site and spindle selection. **a** Nissl stained section taken ~2.76 mm anterior to bregma from an example rat, showing electrode track and recording site (open circle). **b** Unfiltered epochs showing examples of spindles detected for each tree shrews and each rat. Red shaded areas indicate the detected spindle, insets below show the fast Fourier transform (fft) of the detected spindle between 5 and 30 Hz. Cg1 anterior cingulate cortex, M1 primary motor cortex, M2 secondary motor cortex, FMI forceps minor corpus callosum.

Finally, we studied sleep spindles and their interplay with NREM slow waves, since these are considered to be important elements of sleep stability^40–42^ and sleep-dependent memory consolidation^43,44^. Typical delta-band filtered (0.5–4 Hz) LFP or EEG signal segments recorded during NREM are shown for each of the three species in Fig. 5a (top panels). Prominent slow waves are readily discernible in all three species, and could be identified using a standard detection algorithm (see “Methods”). The corresponding spindle-band (10–16 Hz) signal is shown in Fig. 5a (bottom panels), illustrating burst-like spindles that tend to co-occur with slow-waves, consistent with the well-established temporal coupling between these spectral events. We proceeded to examine the distribution of slow wave-spindle coupling events over the time course of the sleep period. Here, we first separately detected slow waves and spindle burst events in the frequency range of 10–16 Hz with a minimal duration of 500 ms (see “Methods” for details), and defined coupling events where a spindle center occurred in a time window of ±2 s centered on the negative slow wave peak. To analyze the distribution of slow wave-spindle coupling events over the time course of NREM sleep, we divided all the NREM epochs of the entire sleep recording into 40 equally sized bins. Subsequently, we computed the relative amount of slow wave-spindle coupling events for each bin. Averaged histograms of each species (see Fig. 5b) reveal significantly higher occurrence of slow wave-spindle couplings during the first eighth of NREM in humans and tree shrews in contrast to rats (paired *t*-tests, *p* < 0.05). To be certain that differences in the polarity of our LFP signals did not impact these results, we inverted the signals for the tree shrew and rat and repeated the analysis. We made three comparisons, inverted rat data vs. inverted tree shrew data, inverted rat data vs. the original, “right side up”, tree shrew data, and inverted tree shrew data vs. right side up rat data. Indeed in all cases the tree shrews showed significantly more coupling early in the sleep period than the rat, (paired *t*-tests, *p* < 0.05). These analyses suggest slow wave to spindle coupling events tend to accumulate during NREM at the very beginning of the sleep period in humans and tree shrews, whereas they tend to be distributed more uniformly throughout the sleep period in rats. In the case of the rats histological evaluation, using Nissl stains, reveal the placement of our electrode in the anterior cingulate, Fig. 6a shows an example. Histological evaluation was not possible in the tree shrews, as this tissue was processed for neuro-peptide analysis. Examples of unfiltered sleep spindles detected by our algorithm are shown in Fig. 6b for both tree shrews and rats, revealing strong spindle events in both species.

## Discussion

We have quantitatively examined sleep architecture in the tree shrew and compared results with findings in rats and humans using similar analytical techniques. During the dark phase of a 12 h/12 h light cycle with gradual illumination transitions, we found that tree shrews were awake only 5% of the time, similar to a previously reported value for this species from a different animal facility (8.8%)^28^. As far as the dark phase is concerned, this suggests that tree shrews exhibit largely continuous sleep with few interrupting wakeful episodes, as is corroborated by the significantly lower number of wakeful episodes in tree shrew compared to rats in the present study. We note that the sleep pattern of the diurnal tree shrew is thus highly similar to the sleep pattern of the slow loris (*Nycticebus javanicus*), as has been observed in a recent field study, although this nocturnal primate displays a reversed activity cycle^45^. Common to both species is the conspicuous absence of wakefulness, or by proxy locomotor activity, in their natural environments during their respective sleep periods. Interestingly, wild slow lorises show largely continuous locomotion during their active period, i.e., the night, although individual animals also do sleep for durations between 20 and 60 min during this period, typically in a single bout. Similar high levels of locomotor activity have also been documented in wild tree shrew, which exhibits quasi-continuous activity during most of the day, with minor daytime resting, presumably sleeping, periods lasting between 40 and 90 min per day that also occur largely in single episodes^29^. Notably, captive tree shrews tend to sleep more extensively during the light period, such that according to one estimate^28^, laboratory tree shrews spend 34% of the light cycle in NREM sleep. It has been suggested that the availability of food and the absence of foraging as well as predation risk explains this discrepancy between wild and laboratory animals^46^. Taken together, we suggest that tree shrew and slow loris, both of which are members of the Euarchonta clade, exhibit a highly similar consolidated sleep structure approximating monophasic sleep, albeit at reversed light cycles.

The large variety in sleep structure across mammalian species can be captured by computational models, which incorporate species-specific parameters for circadian and homeostatic drive. The circadian drive parameter is mostly related to the total sleep duration per day, whereas homeostatic drive is related to sleep fragmentation and captures the accumulation and clearance of somnogens^47^. Species with highly fragmented sleep structure such as rat and mouse possess a homeostatic time constant (*χ*) in the range of 0.2–0.8 h, such that sleep pressure can be reduced at this time scale, explaining the relatively short sleep cycle durations in these species. Species with consolidated monophasic sleep structure, notably primates, possess *χ* parameters of at least 16 h and even up to 45 h^47^, which follows from sleep deprivation studies in these species demonstrating that this time period is necessary for full recovery from sleep deprivation. Indeed, sleep deprivation of tree shrews for a full dark phase also severely impacts sleep during the following light phase, but longer-lasting effects are still evident within the following 12–24 h^28^, suggesting that the *χ* parameter for the tree shrew lies in the same range as for the primate species, i.e., 24–36 h. Convergent evidence thus situate the tree shrew close to primate species in terms of overall sleep structure. This proximity to primates is further supported by our analyses of sleep bout duration distribution, which yielded characteristic τ parameter estimates between 25 and 28 for the tree shrew that is much closer to the value for humans (τ = 22) than for the rat (τ = 6). Note that our data also replicate previously reported τ values in the rat, as well as yielding *α* parameters for brief awakenings that are consistent with previous literature. It has previously been observed that parameters characterizing sleep across mammalian species tend to correlate with physical attributes of the species such as body mass, and this is also the case for both *χ* and τ parameters mentioned above^39,47^. Here, our findings are of considerable scope, as the tree shrew provides an example of a small mammal of under 400 g body mass with characteristic sleep parameter values that far exceed those of similarly sized species. This casts doubt on the idea that body mass is indeed a crucial determinant of mammalian sleep structure. Since tree shrews are phylogenetically close relatives of primates^48^, we consider that consolidated sleep structure may be a shared attribute of scandentia and primates that could be inherited from a common Euarchontan ancestor.

The consolidated sleep during the resting phase in the tree shrew contrasts sharply with the fragmented sleep of the rat, as evidenced by our direct comparisons of sleep cycle duration, number of wake episodes and overall fractions of wake, REM, and NREM. Another important aspect is of course that while tree shrews are diurnal,^49^ rats as well as mice are nocturnal species^50–54^ where sleep occurs mostly during the light phase. However, both mice and rats can, and will, modify their activity patterns depending on environmental exigencies^55,56^. Fragmentation and diurnal/nocturnal status do not necessarily correspond however, as is demonstrated already by the slow loris; a nocturnal primate with low sleep fragmentation as noted above. A notable feature that sets apart human sleep from sleep of many other mammals is the differentiation of NREM sleep into three stages, with NREM1 at the interface between the wake state and slow-wave sleep^57,58^ and characterized by mixed theta and alpha band activity. Here, we document in the tree shrew a transition state, IS-NREM, from NREM to REM sleep, which occurs during periods of decreasing delta power and displays a clear peak in alpha-theta activity. The IS-NREM sleep stage of the tree shrew shares the spectral signatures of NREM1 in humans, and is clearly separable from NREM, with high delta power and absence of enhancement in the theta alpha range. However, it characterizes transitions from NREM to REM sleep as opposed to human NREM1 which occurs on wake to sleep transitions. Our quantitative analyses of the IS-NREM to REM transitions show that the IS-NREM is highly reproducible across transition instances and individual animals in our intracranial ACC recordings in the tree shrew. While we did not observe an intermediate stage that included increased alpha/theta activity during NREM-REM transitions in the rat, such intermediate states have been previously observed^9,10^. This discrepancy may be due to differences in data scoring. While we used epochs of 10 s duration for sleep analyses, previous studies that have observed intermediate state activity in the rat have used 4 s epochs,^9^ or visual scoring^10^. Indeed identification of sleep states is known to be highly dependent on analytical techniques^59^. We used a 10 s epoch length here to facilitate cross-species comparison, and in this sense our findings underline the dissimilarities between rat and tree shrew sleep structure. An interesting aspect of tree shrew IS-NREM is that it appears to occur unidirectionally for transitions from NREM, to REM, which suggests that it might be related to neural circuit activation involved in REM initiation. REM initiation has been linked to circuits in the brain stem and medulla^60,61^, such that for example activation of GABAergic neurons in the ventral medulla triggers transitions into REM state^8^. We suggest therefore that the peaks in theta-alpha activity observed during the IS-NREM transitions in the tree shrew may provide one possible signature of activations of these REM initiating circuits. Our analyses of human EEG data revealed that a substantial proportion of NREM-REM transitions in humans were also accompanied by elevated theta-alpha activity, suggesting a degree of homology between tree shrews and humans. However, no theta-alpha activity was observed in the majority of transitions in the human EEG, an effect that could reflect the regional local sleep occurrence that has been described in the human brain^62,63^, with effects of epoch duration or differences between surface and intracranial electrophysiological recordings as other potential sources for this variability. While we highlight evidence for homology, there were also obvious species-related differences between human and tree shrew sleep, including a shorter sleep cycle and more waking periods in tree shrews as compared to humans. Tree shrews thus represent an intermediate between primates and rodents in terms of sleep, and therefore provide an important model for comparative sleep studies. It is well established that memory formation is strongly impacted by sleep. For example, studies in both rats and mice have shown that sleep deprivation negatively impacts novelty preference in both novel object and novel location tasks^64–67^. Available evidence suggests that sleep is most effective when it occurs directly after task performance^68^, such that many authors investigating sleep indeed choose to conduct behavioral encoding sessions during the inactive, resting period in rodents to maximize the impact of sleep-dependent memory consolidation. Similar findings have been reported in human subjects. For example, the performance of a visual perceptual learning paradigm improves across days for subjects that had access to overnight natural sleep but not subjects that were sleep deprived^69^. Sleep is composed of REM and NREM sleep. Convergent evidence in rats and humans points to important and possibly dissociable roles of both REM and NREM sleep phases in memory formation^70–73^. We have documented that sleep spindles are present during NREM sleep with significant coupling to slow oscillation delta waves in all three species investigated. Slow wave to spindle coupling events have been extensively documented in humans, where a link to memory consolidation has been uncovered in the sense that coupling events occur more frequently in sleep epochs following learning tasks compared to control periods^31,38,74^. A role for spindles coupled to slow waves in memory formation is also corroborated by findings in rodents, where triggering of spindles around the occurrence of slow waves enhanced memory formation^36^. Our findings present, to our knowledge, the first comparison of the dynamics of slow wave to spindle coupling events throughout the sleep period. Interestingly, coupling events tended to occur predominantly at the start of the sleep period in tree shrews and humans, which are the two species with consolidated sleep structure. In rats, coupling events were distributed more equally throughout the sleep period, consistent with the stronger fragmentation of rodent sleep described in detail also above. While not examined in this study, we speculate that the concentration of coupling events in tree shrews may contribute to the particularly robust formation of long-lasting memories in this species, for example in novelty tasks^20,21^ or in fear conditioning paradigm where retention periods can exceed 50 days^75^.

## Methods

### Surgical procedures

The local ethical committee on animal experimentation (canton of Fribourg), approved all experimental procedures.

### Rats

Five adult male Long Evans rats with an average age of 31.7 (SEM = 0.9) weeks were used in this study. Note that the rats used in this study were of ages that roughly corresponded to our tree shrew subjects, and while age-related changes in sleep are a concern, this does not seem to occur in rats until they are significantly older than those used in this study,^76^. Rats were single housed under a constant 12/12-h light/dark cycle with access to food and water ad libitum. For surgeries, animals were anesthetized with ketamine (100 mg/kg, i.p.) and xylazine (20 mg/kg, i.p.), and anesthesia was maintained using isoflurane (around 2%) in pure O2 inhalation. We implanted four tungsten microelectrodes (FHC Inc. Bowdoin ME, USA), with ~400 kΩ impedance 1 in left and 1 in right ACC (AP 3 mm, ML ±0.8 mm, DV − 3.0 mm from bregma), and 1 in left and 1 in the right basal forebrain (AP -0.8 mm, ML ±2.8 mm, DV − 8.0 mm from bregma). A stainless-steel screw above the cerebellum served as the reference and ground for electrodes. Electrodes and EEG screws were wired to a customized 10 pin connector/PCB board attached to a ziff clip head stage (TDT Apalucha, FL). The connector and leads were fixed to the animal’s skull and stabilized with five stainless steel screws and dental cement. Animals were administered a single post-operative dose of Buprenorhpine (0.05 mg/Kg, SC) and Vetramil ointment was applied to the margins of the skin incision. Animals received postoperative analgesia in the form of Paracetamol dissolved in drinking water (2 mg/ml) for 3–4 days following the surgery. Sleep recordings were conducted >1 week following electrode implantation. Following the experiments rats participated in an additional study, and were then deeply anesthetized, pentobarbital 100 mg/kg i.p., and perfused transcardially with 500 ml PBS followed by 500 ml 4% paraformaldehyde.

### Tree shrews

For this study we used four adult Tree Shrews (two males and two virgin females) (Tupaia Belangeri, 6–12 months old). Virgin female tree shrews were isolated from males in order to avoid confounds due to hormonal fluctuations, as under these conditions animals do not enter estrous^77^. However, as the number of subjects is limited, we cannot eliminate the possible contribution of gender to the observed results. The animals were maintained at 13/11 light/dark cycle with gradual illumination transitions at the beginning and end of the light period. Food and water were available ad libitum. For surgeries, we induced anesthesia with ketamine (200 mg/kg, IM injection) and xylazine (5 mg/kg, IM injection) and maintained with 1–2% isoflurane in pure O2. After shaving the scalp, the skull was exposed with a midline incision. A small bur hole was made at the location of the left ACC, AP 11.1, ML 0.8, DV 1.5, from the interaural line. A single tungsten microelectrode (FHC Inc. Bowdoin USA, tip resistance around of ~400 KΩ) was then lowered to the target, and anchored with dental cement. The electrode was tied to a miniature connector with flexible cables, also fixed to the animal’s skull. Two to three stainless steel anchoring screws were used to stabilize the implant with one screw over the cerebellum serving as a reference. The margins of the incision were closed with resorbable Vicryl suture, and the animals received postoperative analgesia in the form of Buprenorphine, 0.05 mg/kg, IM. Prior to their release from the nest box, animals were closely monitored until they woke up from the anesthesia. In order to prevent infections, the skin around the implant was treated topically with an antibacterial ointment and cleaned with Betadine whenever needed. All recordings took place at least one week after electrode implantation. Following the sleep experiments, tree shrews were sedated with isoflurane, and euthanized by rapid decapitation.

### Data acquisition rats

A plastic box (30 × 50 × 40 cm) with nesting material and a drinking bottle attached placed in a noise attenuated chamber, served as a home cage-like environment for recording sessions. We conducted 8 h sleep recordings during the middle of the light cycle (7 am to 7 pm) between 8:30 am to 6 pm. A camera was mounted 45 cm above the floor of the box. Tethered recordings were conducted by using a motorized commutator (ACO32, TDT Alachua, FL). LFP data were acquired through a unity gain head stage (TDT Alachua, FL) and was sampled at 2.4 kHz and band pass filtered between 0.5 and 300 Hz using an RZ5 amplifier (TDT) and stored on a PC for offline analysis.

### Data acquisition tree shrews

Tree shrews were housed in 6 m^3^ cages with a wooden nest box attached to the main body of the cage. All recordings were 8 h long, and were acquired during the dark phase, 19:30–03:30, in the nest box where the animals spent the night sleeping. We recorded activity with a sampling frequency of 500 Hz using a miniature wireless data logger (Neurologger 2A, Zurich Switzerland) equipped with a 3-D accelerometer to monitor movement.

### Data acquisition humans

Five healthy human subjects were included (three females and two males) with an age range of 20–22 years (mean = 21, SEM = 0.5 years). Undergraduate students of Psychology were recruited by E-mail or from the campus of the University of Fribourg through advertisements. Before participation, subjects had to give written informed consent as approved by the Ethical Commission of the Department of Psychology of the University of Fribourg. The participants were instructed to keep a normal sleep schedule, to get up in the morning before 8 am and not to consume alcohol and caffeine on the study day. For participation, they either received credit for an undergraduate class and/or monetary compensation.

As for the rats and tree shrews, sleep recordings were 8 h long and were conducted from 11 pm until 7 am in the sleep laboratory of the University of Fribourg. We made EEG recordings by using customized 32-Ag/AgCl electrodes at 10-10 locations caps (EASYCAP) and 32 channel amplifiers (Brain Products). Impedances were kept below 10 kΩ. The EEG was recorded with a sampling rate of 500 Hz using Brain Vision Recorder software (Brain Products). Signals were referenced to electrodes at the mastoids. We conducted all analyses based on the Fz electrode over the midline prefrontal cortex in order to approximate the ACC recording location used in the rats and tree shrews. The ocular activity was measured via one EOG channel mounted ~2 cm below the right eye. Muscle tone was monitored by EMG recordings made under the chin.

### Adaptation to the test environment

“Rats were allowed to acclimatize to the sleep arena and the tether used for data acquisition for a period of one week. Tree shrews were habituated to the wireless recording device, also over the period of one week, and otherwise required no further familiarization sessions as they were sleeping in their own nest box. The human subjects had no additional habituation, except for an adaptation period prior to the night where data were collected.”

### Data processing and statistical analysis

All data were processed offline and analyzed using custom-made MATLAB scripts and functions.

### Sleep scoring in tree shrews and rats

In rats and tree shrews, we manually scored the behaviors wake, NREM, and REM sleep based on 10 s epochs by using custom software written in MATLAB. In rats, a movement signal trace was extracted from the video data for the specific epoch by computing the averaged frame to frame difference over time and over all pixels, absence of movement, closed eyes, and a sleep like posture discriminated sleep from wake. In tree shrews, movement signals were monitored using the accelerometer on the Neurologger device. While the accelerometer does not distinguish between REM and NREM during stable periods, transient increases in movement sensor signals are observable at the interfaces between sleep stages or sleep and wakefulness. The absence of movement and the presence of slow waves and sleep spindles indicated NREM sleep. REM sleep was defined as a combination of flat movement signals, the absence of slow waves and sleep spindles, and pronounced theta band activity. We scored wake based on movement signal fluctuations and a desynchronized LFP signal with gamma activity (30–80 Hz) in the power spectrum.

In tree shrews, recordings were re-scored after initial sleep scoring to further divide NREM sleep into an intermediate stage, IS-NREM, and NREM. IS-NREM epochs were scored during transitions based on observations of pronounced theta-alpha activity, medium delta wave activity, and flat movement signals. For humans, Sleep stages NREM1, NREM2, NREM3, wake, and REM sleep were scored manually according to the criteria of the American Academy of Sleep Medicine by monitoring signals of frontal, central, and occipital electrodes over 30 s epochs.

### Sleep spindle detection

Sleep spindles were detected by detrending and bandpass filtering (9–16 Hz) the LFP signal. The amplitude threshold was calculated individually for each subject and session as 1.5 SDs from the mean. Spindle beginning and end times were then defined as the points at which the amplitude fell below 1.5 SDs before and after the detected spindle. Only sleep spindles with a duration of 0.5–3 s and a spindle frequency of >10 Hz were included in subsequent analyses.

### Slow-wave detection

To detect slow waves over all three species, we first localized all negative and positive peaks of the 0.5–4 Hz band-pass filtered signal during NREM sleep epochs. For the initial detection of the slow waves we used the following inclusion criteria: (1) The positive peak prior to the negative peak should be >0.5 SDs of the filtered signal (0.5–4 Hz); (2) the negative peak should be smaller than −0.5 SDs and (3) the time distance between the prior and the posterior positive peaks should be in the range of 0.5–2 s.

### Slow wave-spindle coupling analyses

Based on detected slow waves and sleep spindles, we defined coupling events when the center of a detected sleep spindle was occurring in a time window of ±2 s centered on the negative slow wave peak. To analyze the distribution of slow wave-spindle coupling events over the time course of NREM sleep, we divided all the NREM epochs in 40 equally sized bins. Subsequently, we computed the relative amount of slow wave-spindle coupling events for each bin. Statistical comparisons between species were conducted based on the first eighth of NREM sleep by summation of the first five bins per subject (see Fig. 3b). One tree shrew recording revealed longer epochs of corrupted electrophysiological data by artefacts and was excluded from this analysis.

### LFP power analyses

For the power spectrograms (see Figs. 1a, d; 2a and 3a–c at upper panels), the LFP data were down-sampled to 500 Hz and fast Fourier transform with log10-scale was computed for 10 s data epochs. Finally, power data was plotted over time with a frequency resolution of 0.5 Hz. To calculate the delta (0.5 to Hz) and theta-alpha (4–12 Hz) power over time, a fast Fourier transform was performed for 10 s epochs and the averaged power values were computed over the two frequency ranges of interest per epoch. Next, we conducted a sliding average (step-size of 1 and a cantered time window of 10 epochs) over the time series of power values (see Figs. 1a and 2a and 3a–c at lower panels).

### Transition analyses

We used the first derivative of the delta power time series (see methods above LFP power analyses), to identify the midpoint transition times by finding the negative peaks between zero-crossings, where peaks were defined as epochs where the derivative became negative to −0.3 d(Δpower)/dt. We then calculated the theta-alpha power at three points before, during, and after these peak times, corresponding to NREM, transition, and REM. NREM: −25 to −10 s, transition: −5 to +5 s, and REM: +10 to +20 s. Statistical comparisons for these different states are shown for tree shrews and rats in Fig. 1c, f. In humans, transition segments were manually selected by visual inspection of the delta power traces of the recording periods with respect to greater diversity in human sleep transitions. Examples of the analyzed segments in humans are shown in Fig. 2.

### Sleep cycle identification

In order to quantify the sleep cycles per hour (Fig. 4a), sleep cycles were defined as the time range between the start of an NREM episode and the end of the following REM episode. The count of wake bouts was computed from the sleep annotation after sleep onset (Fig. 4b).

### Distribution of sleep bout durations

We analyzed the distribution of sleep bout durations for rats and tree shrews. A pooled sample of sleep durations for rats and tree shrews were selected. The cumulative probability distribution was then calculated for the samples of the two species. On a semi-logarithmic scale, both datasets display a decrease following a straight line (see Fig. 4d). The lines in Fig. 4d show the fitted power law function for each dataset with their characteristic time constant τ calculated as shown in a previous publication^47^. The insert in Fig. 4d was adapted from a previous work^39^ with data points maintained of mouse, cat and human. Data points of tree shrew and rat were added from our own analysis.

### Statistical analyses

Paired t-tests were used to compare data within animals. To conduct between group analyses, we used unpaired *t*-tests. Comparison analyses between percentage values of sleep stages were performed by using *χ*^2^-tests. All data were analyzed by using MATLAB 2018a. Data are presented as the mean ± SEM and statistical significance is considered at *p* < 0.05. To standardize data with respect to individual differences, Z-score values were computed over the distribution of measurement points within animals (Fig. 1c, f). All data used in the preparation of the figures are available in supplementary data 1.

### Reporting summary

Further information on research design is available in the Nature Research Reporting Summary linked to this article.

## Supplementary information

Description of Additional Supplementary Files

Supplementary Data 1

Reporting Summary

## Data Availability

The data that support the findings of this study are available from the corresponding author upon reasonable request.
