# Supplementary material for: Aspects of tree shrew consolidated sleep structure resemble human sleep
Source: Commun Biol. 2021 Jun 11;4:722. doi: 10.1038/s42003-021-02234-7 (PMC8196209; doi:10.1038/s42003-021-02234-7)
Supplement: Supplementary file 1 — Description of Additional Supplementary Files [file 42003_2021_2234_MOESM1_ESM.pdf]

Description of Additional Supplementary Files

File Name: Supplementary Data 1
Description: Figure source data.
