# Supplementary material for: Aspects of tree shrew consolidated sleep structure resemble human sleep
Source: Commun Biol. 2021 Jun 11;4:722. doi: 10.1038/s42003-021-02234-7 (PMC8196209; doi:10.1038/s42003-021-02234-7)
Supplement: Supplementary file 3 — Reporting Summary [file 42003_2021_2234_MOESM3_ESM.pdf]

nature research

Corresponding author(s): Gregor Rainer

Last updated by author(s): 2021/04/28

Reporting Summary

Nature Research wishes to improve the reproducibility of the work that we publish. This form provides structure for consistency and transparency in reporting. For further information on Nature Research policies, see our Editorial Policies and the Editorial Policy Checklist.

Statistics

For all statistical analyses, confirm that the following items are present in the figure legend, table legend, main text, or Methods section.

n/a	Confirmed	
[ ]	[x]	The exact sample size (*n*) for each experimental group/condition, given as a discrete number and unit of measurement
[x]	[ ]	A statement on whether measurements were taken from distinct samples or whether the same sample was measured repeatedly
[ ]	[x]	The statistical test(s) used AND whether they are one- or two-sided<br>*Only common tests should be described solely by name; describe more complex techniques in the Methods section.*
[x]	[ ]	A description of all covariates tested
[x]	[ ]	A description of any assumptions or corrections, such as tests of normality and adjustment for multiple comparisons
[ ]	[x]	A full description of the statistical parameters including central tendency (e.g. means) or other basic estimates (e.g. regression coefficient) AND variation (e.g. standard deviation) or associated estimates of uncertainty (e.g. confidence intervals)
[ ]	[x]	For null hypothesis testing, the test statistic (e.g. *F*, *t*, *r*) with confidence intervals, effect sizes, degrees of freedom and *P* value noted<br>*Give P values as exact values whenever suitable.*
[x]	[ ]	For Bayesian analysis, information on the choice of priors and Markov chain Monte Carlo settings
[x]	[ ]	For hierarchical and complex designs, identification of the appropriate level for tests and full reporting of outcomes
[x]	[ ]	Estimates of effect sizes (e.g. Cohen's *d*, Pearson's *r*), indicating how they were calculated

Our web collection on statistics for biologists contains articles on many of the points above.

Software and code

Policy information about availability of computer code

Data collection	Humand EEG data were acquired using Brain Vision Recorder software (Brain Products, Morrisville, NC);Tree Shrew data was acquired using Multichannel Experimenter (Multichannel Systems, Reutlingen, Germany), and Rat data were acquired using OpenEx(TDT, Apalucha, FL)
Data analysis	All data were analyzed using Matlab 2017a (Mathworks, Natick MA).

For manuscripts utilizing custom algorithms or software that are central to the research but not yet described in published literature, software must be made available to editors and reviewers. We strongly encourage code deposition in a community repository (e.g. GitHub). See the Nature Research guidelines for submitting code & software for further information.

Data

Policy information about availability of data

All manuscripts must include a data availability statement. This statement should provide the following information, where applicable:

- Accession codes, unique identifiers, or web links for publicly available datasets
- A list of figures that have associated raw data
- A description of any restrictions on data availability

The data used in this study are available from the corresponding author upon reasonable request.
author upon reasonable request.

Field-specific reporting

Please select the one below that is the best fit for your research. If you are not sure, read the appropriate sections before making your selection.

☒ Life sciences ☐ Behavioural & social sciences ☐ Ecological, evolutionary & environmental sciences

For a reference copy of the document with all sections, see nature.com/documents/nr-reporting-summary-flat.pdf

Life sciences study design

All studies must disclose on these points even when the disclosure is negative.

Sample size	Sample sizes were determined based on availability of subjects in the case of Tree shrew, in the case of rats and humans samples sizes were selected based on existing literature and laboratory experience.
Data exclusions	We excluded some data from one tree shrew where electrophysiological recordings were corrupted. This was based on examination of the data at the time of analysis.
Replication	We have not replicated this data.
Randomization	There were no treatment groups.
Blinding	As there were no treatment groups, apart from the use of different species, blinding was not possible.

Reporting for specific materials, systems and methods

We require information from authors about some types of materials, experimental systems and methods used in many studies. Here, indicate whether each material, system or method listed is relevant to your study. If you are not sure if a list item applies to your research, read the appropriate section before selecting a response.

Materials & experimental systems

n/a	Involved in the study	
☒	☐	Antibodies
☒	☐	Eukaryotic cell lines
☒	☐	Palaeontology and archaeology
☐	☒	Animals and other organisms
☐	☒	Human research participants
☒	☐	Clinical data
☒	☐	Dual use research of concern

Methods

n/a	Involved in the study	
☒	☐	ChIP-seq
☒	☐	Flow cytometry
☒	☐	MRI-based neuroimaging

Animals and other organisms

Policy information about studies involving animals; ARRIVE guidelines recommended for reporting animal research

Laboratory animals	Rats: Rattus norvegicus; Long Evans N=5 (5 males). Tree Shrews: Tupaia belangeri, N=4 (2 males;2 females)
Wild animals	No Wild animals were used
Field-collected samples	No samples were collected in the field.
Ethics oversight	Laboratory Animal ethical approval was obtained from the Local Ethical Committee on Animal Experimentation (Canton of Fribourg, Switzerland). Human subject approval was granted by the Ethical Commission of the Department of Psychology of the University of Fribourg.

Note that full information on the approval of the study protocol must also be provided in the manuscript.

Human research participants

Policy information about studies involving human research participants

Population characteristics	5 healthy human subjects between the ages of 20 22 years were studied. There were 3 female and 2 male participants.
Recruitment	Undergraduate students of Psychology were recruited by E-mail or from the campus of the University of Fribourg through advertisements.

Ethics oversight	Local Ethical Committee on Animal Experimentation (canton of Fribourg, Switzerland).<br>Ethical Commission of the Department of Psychology of the University of Fribourg.

Note that full information on the approval of the study protocol must also be provided in the manuscript.
